# Orthopantomography contribution to prevent isquemic stroke

**DOI:** 10.4317/jced.51352

**Published:** 2014-04-01

**Authors:** Pedro Abecasis, Eduardo Chimenos-Küstner, osé López-López

**Affiliations:** 1 Phd in Odontology. Profesor of Oral radiology in ISC-Egas Moniz. University of Odontology, Portugal; 2 Phd in Medicine and Surgery. Profesor Oral Medicine, Facultad de Odontología, Universidad de Barcelona

## Abstract

Objectives: The ortopantomography (OPG) can be a valuable way for an early detection of calcified atheroma plaques, thus contributing for a preliminary stroke risk evaluation. The study looks for the existence of calcified atheroma plates through the use of OPG, comparing the results with the stenosis percentage found through eco-doppler. It has been analyzed the correlation of the number of years as a smoker, arterial hypertension and body mass index, against the risk of having calcified atheroma plaques. Study Design: Observational, transversal and prospective study with 84 patients from the Dental Center of Hospital Particular de Lisboa. First the patients answered to an inquiry and them they were submitted to an OPG and an eco-doppler. Results and Conclusions: It is possible to detect calcified atheroma plaques in the carotid artery through an OPG and patients who have them have got a fifteen fold greater risk of suffering from carotid stenosis. In this study, it has been confirmed the increase in carotid stenosis for long term smokers (OR = 1,033, n=18, 42,9%). The study results show that hypertension patients have a probability 5,426 greater than normal of developing atheroma plaques (with sig=0,049). Amid analyzed patients, the correlation between obesity and the existence of carotid atheroma plaques was significant, although negative (sig=0,047). OPG can help find patients with higher risk of isquemic stroke.

** Key words:**Orthopantomography, Stroke, Carotid disease, Calcified atheroma.

## Introduction

A CVA (cerebrovascular accident) is defined as a sudden loss of brain function due to ischemia or hemorrhaging in the central nervous system. In Spain, the mortality rate associated with CVAs has decreased in recent decades, but it is still the second-highest cause of death among men and the first in women (1). According to Perkins (2), in 2010, 75% of CVAs were caused by ischemia and 20-30% originated in the carotid bifurcation.

There are various significant risk factors to consider regarding carotid atherosclerosis. Age is one such factor, due to both the cellular changes inherent in the aging process and great exposure to other risk factors, such as tobacco, hypertension, cholesterol and obesity (2). Also, obese individuals are more susceptible to carotid dis-ease (3).

Additionally, tobacco use is an important independent risk factor for CVAs (increasing the risk by up to six times) (4). Arterial hypertension is the most important and prevalent modifiable risk factor for CVAs (5).

Orthopantomographs (OPGs) are frequently used in dentistry and offer a general view of patients’ upper and lower teeth; they also provide information about the temporomandibular joints and the cervical vertebrae (6,7). In 1984, Friedlander (8) conducted a study with 295 patients over the age of 55, in which 3% presented calcified atheromatous plaques in the OPGs. The same author, in a more recent publication, correlates the OPG with the clinical value of the diagonal earlobe crease (9). Carter (10), in 1997, identified calcified atheromatous plaques in the OPGs of 3.6% of a sample of 1,175 male and female patients with an average age of 40 years. In that study, only the correlation with obesity was statistically significant.

In a study of 11,854 patients carried out in Rotterdam by the Bots (11) group, it was concluded that patients with carotid disease are at an increased risk of having an ischemic CVA.

When using radiological imaging, anatomical structures such as the triticeous cartilage, and the stylomandibular and stylohyoid ligaments, which, if calcified, will contribute to the differential diagnosis, must always be consi-dered. There are also pathological masses such as salivary calculi or calcified lymph nodes which must be known in order to differentiate them from the aforementioned structures.

Carotid stenosis is directly related with cerebrovascular accidents, and when they are due to calcified athermatous plaques, it is possible to detect them on the orthopantomography (OPG). Based on this, we aim to study the correlation between the presence of calcified plaques on the OPG and the degree of stenosis. From a cardiovascular point of view, we know that stenosis greater than 70% is considered to be high and is closely correlated with an increased risk of CVA (12).

The carotid bifurcation shows hemodynamic changes and a reduction in nitric oxide production, which leads to an increase in vascular damage. The nitric oxide produced in the endothelium regulates different mechanisms such as vasodilatation, inhibition of activation, adhesion and plaque buildup, and also participates in the regulation of basal blood pressure (13). Individuals suffering from stenosis higher than 70%, reduce their risk of a CVA by undergoing surgery (13).

## Objectives

To detect the presence of calcified atherormatous plaques in the carotid artery using OPG and their correlation with stenosis found using eco-doppler.

To relate the results obtained with age, number of years of tobacco use, obesity and hypertension.

## Material and Methods

An observational, longitudinal and prospective study is carried out with patients of the Servicio de Medicina Dentária (Dentistry Unit) at the Hospital Particular de Lisboa in Lisbon, Portugal. The project has been approved by the Comité Ético de Investigación Clínica del Hospital Particular de Lisboa (Clinical Research Ethics Committee of the Hospital Particular de Lisboa).

After a pilot study for a 95% test power and in accordance with the relevant literature for calculating the sample sizes, the number 42 was arrived at for the patients in each group (42 people with atheromatous plaques in the OPGs and a control group of 42 patients without atheromatous plaques in the OPGs).

Patients were selected upon their arrival to the dental medicine office by a dentist who evaluated the OPGs of 1,176 patients (from 2008 to 2012) until finding 42 patients with the presence of calcified atheromatous plaques in their OPGs.

The OPGS of all the patients were taken using a Kodak 8000C Digital panoramic and cephalometric system machine (Kodak, 2007).

To carry out the study, a questionnaire was prepared (which evaluated if the patients had hypertension, were smokers and for how long, their weight and height). A form explaining the study was provided to them and their consent was requested.

The first 42 patients who met the requirements (older than 60, born in Portugal, excluding those who suffered from a previously diagnosed vascular pathology or who had previously undergone carotid surgery) made up the control group.

The duration of the radiographic examination was 14 seconds, with a 6mA current and an 85 kV voltage. An eco-doppler test was later done (using a Logiq-500 General Electric model) on all 84 patients to evaluate carotid flow, determining the percentage of stenosis in those such cases. This examination was done in the Servicio de Cirugía Vascular (Vascular Surgery Unit) in the same hospital.

The percentage of stenosis for each patient was entered into the data spreadsheet (using Microsoft Excel 2007), and then statistical analysis of the questionnaire responses and the results was performed using SPSS version 15.0.

All OPGs were analyzed by the same dentist, looking for the presence of atheromas in the area of carotid bifurcation (C3-C4) and examining circular or vertical line radiopacities, to differentiate between the OPGs with and without atheromas. The dentist analyzed the x-rays using a computer, focusing on the bifurcation of the carotid arteries, 2 to 4 cm below the mandibular angle.

For studying obesity, the universal body mass index formula was used, and patients with a value equal to or greater than 30 were considered obese.

## Results

Of 1,142 orthopantomographs, 42 showed plaques; in other words, 3.68% of the x-rays presented images com-patible with calcified atheroma.

The correlation between having atheromatous plaques and hypertension was significant (*p*=0.049, phi coefficient=0.214 using chi-square). 61.9% of the participants who presented calcified atheromatous plaques in the OPGs had diagnosed hypertension ( Table 1).

**Table 1:**
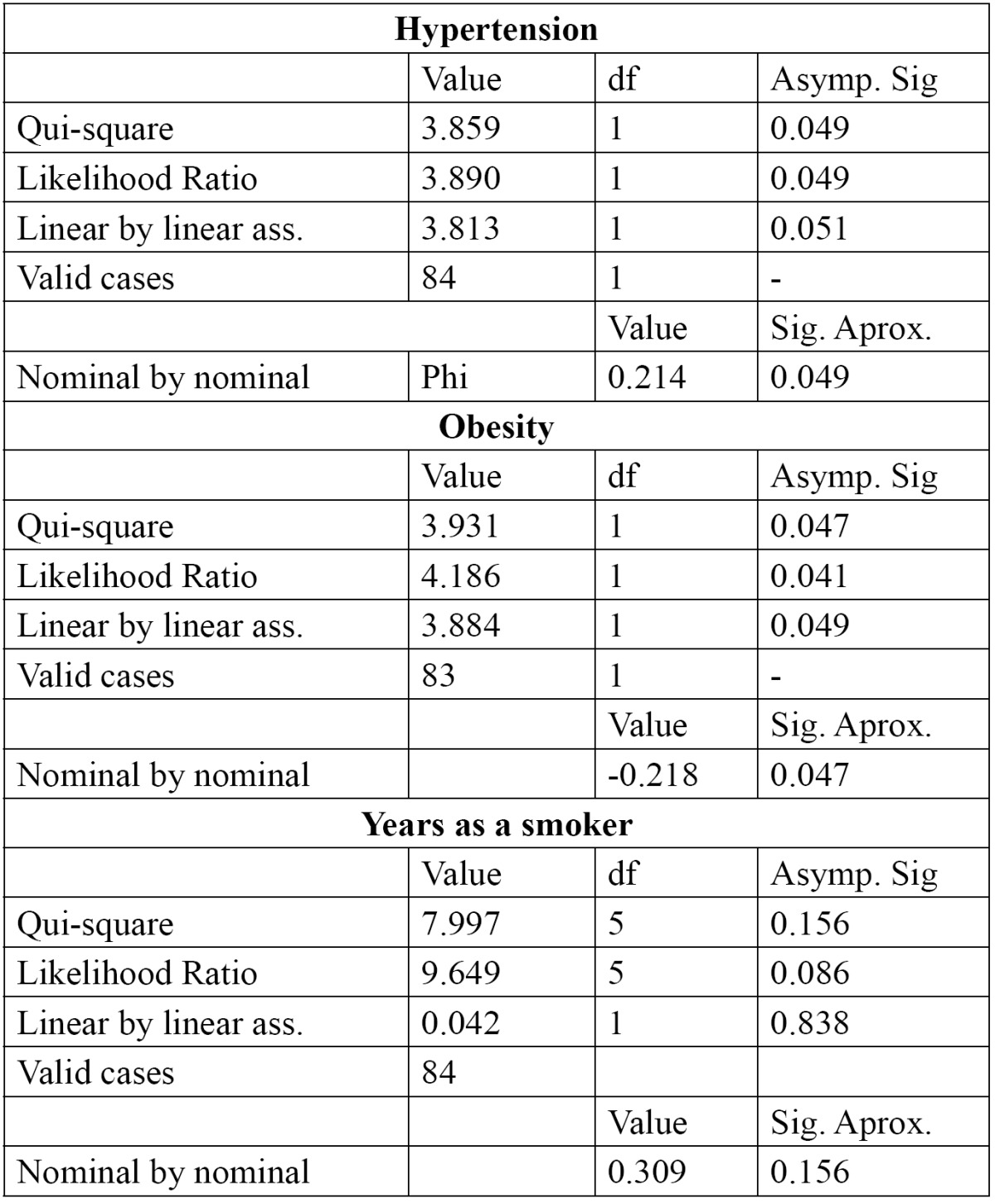
Correlation between hypertension, obesity, number of years as a smoker and to have atheromas.

The correlation between having plaques and obesity was significant (*p*=0.047), but in a negative direction (phi=-0.218). In the group with atheromatous plaques, there are less patients with obesity (n=3) than in the group without atheromatous plaques (n=8) ( Table 1).

When studying the correlation between having plaques and the number of years of smoking tobacco, it is worth noting that there are more smoking participants without plaques in the group (n=25, 59.5%), but in the group with plaques, the participants who smoked (n=18, 42.9%) had been doing so for more years. There is a significant correlation between having plaques and the number of years of smoking (*p*=0.156 and phi=0.309) ( Table 1).

A stepwise logistic regression was performed to study the correlated risk factors, and using the Wald test it was determined that the most significant coefficients are age (*p*=0.034 and OR=1.114) and hypertension (*p*=0.031 and OR=5.246) ( Table 2).

**Table 2:**
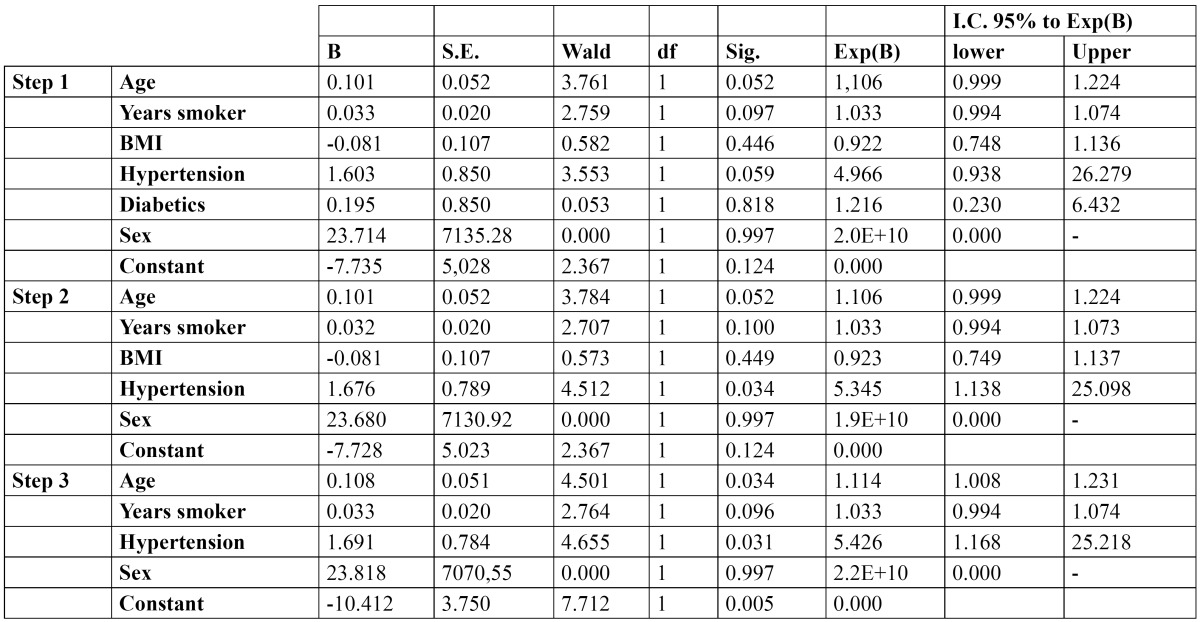
Results of the logistic regresion.

It was determined that there is a correlation between having atheromatous plaques visible on the OPG and the percentage of stenosis on the eco-doppler, with a confidence interval of 95%. The group with plaques on the OPG had a risk 15 times greater of stenosis greater than 70% on the eco-doppler ( Table 3). Of the 42 patients who presented atheromas on the OPG, 15 had a high degree of stenosis (greater than 70%), while of the group without visible plaques on the OPG, only one had a high degree of stenosis.

**Table 3:**
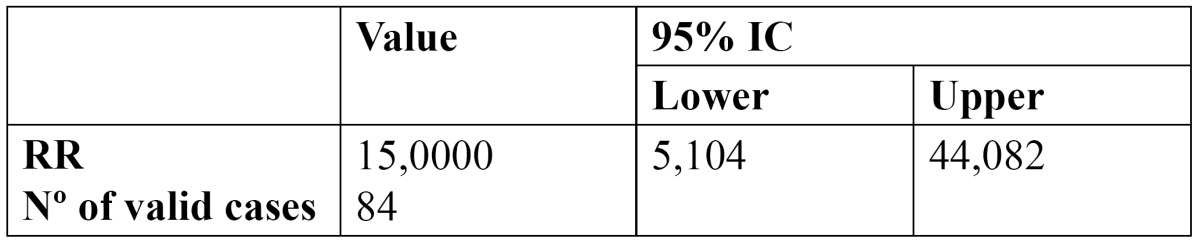
Relative risk.

## Discussion

Some authors indicate that the age 55 and over population is the one with the highest risk of atheromatous plaques (14). In this study, the risk of appearance of atheromatous plaques in the carotid artery increases by 1.114 for each year that patients live after the age of 65.

Hypertension is the most predictive factor for CVAs (15). In this study, a patient with hypertension is 5.426 times more likely to have plaques, and when studied independently, hypertension is significantly correlated (sig=0.049 and phi=0.214).

The positive correlation between calcifications in the carotid artery and smoking is also identified (16,17). In this study, prolonged tobacco exposure is confirmed as an increasing factor of carotid stenosis, increasing 1.03 times for each additional year of smoking, and when studied independently, the number of years smoking is significantly correlated (sig=0.156) and phi=0.309).

While obesity is positively correlated with the presence of calcified atheroma (8), we have not obtained conclusive results in this study, possibly because of the nature of the sample, since the direction of the correlation turned out to be negative.

There are many studies that speak of obesity as a risk factor (4) for cardiovascular disease. There is also scientific evidence defending the paradox of obesity, according to which obesity functions as a protective factor and not as a risk factor. Thus, for example, Hastie (18) mentions some hypotheses for justifying why obese patients who undergo coronary bypass have a better prognosis when compared with non-obese patients. Obese patients are medicated earlier, suffer vascular accidents (such as heart attacks) at younger ages, making age a benefit; it is very probable that body mass index is not the best way to measure obesity, since it does not distinguish between different types of obesity (amounts of fat or muscle mass). Stephan (19) states that obese patients retain many TNF-α receptors in their fat deposits, which may cause a reduction in circulation, reducing atherogenic activity in vascular diseases. It is important to study what percentage of the population is aware of the problem of atherosclerosis and the risk factors. Awareness and prevention campaigns should be organized in light of this study, which shows that the existence of calcified atheromatous plaques in the carotid artery represented a 15 times greater risk of stenosis compared to the group without calcified plaques in the OPGs.

It is crucial to always obtain a detailed clinical history to learn about patients’ harmful habits and any prior pathological, personal and family events.

In conclusion, we have stated taht identifying calcified atheromatous plaques using panoramic x-rays may detect a specific risk of a CVA when used as a secondary instrument (1). Therefore, the examinations performed daily by dentists show more structures than just the teeth. It is thus very important that dentists are aware of this and refer to specialists those patients who may present a pathology like the one described. Calcifications in the carotid arteries are a marker of advanced atherosclerotic disease and a predictor of events.

Dentists are in a unique position; given that they can perform a non-invasive examination such as the OPG, they can contribute to reducing mortality due to CVA.
